# Occupational Health Nurses in European Nuclear Power Plants: Safety, Readiness, and Well-Being Require a Heuristic Training Framework

**DOI:** 10.7759/cureus.111368

**Published:** 2026-06-23

**Authors:** Giuseppe Fumai, Francesco Menolascina, Claudio Morelli

**Affiliations:** 1 Orthopedic and Neurological Rehabilitation, Rehcura Rehabilitation Facility, Adelfia, ITA; 2 Nursing, Azienda Sanitaria Locale di Bari (ASL Bari), Bari, ITA; 3 Nursing, Istituto di Ricovero e Cura a Carattere Scientifico (IRCCS) Istituto Tumori Bari, Bari, ITA

**Keywords:** emergency preparedness, europe, ionizing radiation, nuclear power plants, nursing, occupational health nursing, occupation safety, organizational well-being, radiation protection, willingness to respond

## Abstract

Across Europe, nuclear power plants operate within sophisticated regulatory systems that tightly govern occupational radiation exposure. International and European frameworks, the International Atomic Energy Agency (IAEA) Safety Standards and the Basic Safety Standards Directive 2013/59/Euratom, define robust requirements for monitoring, dose optimization, and worker protection, yet translate only weakly into nursing-specific competencies and educational pathways. Available evidence suggests that, where it has been assessed, emergency nurses’ knowledge of radiation protection and response is poor; that willingness to respond is shaped more by psychological and occupational factors than by technical knowledge alone; and that long-term, low-dose occupational radiation exposure is associated with measurable job stress and burnout among medical staff. Yet radiation-specific competencies remain underdeveloped in nursing education. This editorial argues that European specialist nursing education and continuing professional development should incorporate a structured, radiation-focused training framework for occupational health nurses working in or with nuclear power plants. We propose a heuristic model, presented as a practice-oriented table rather than a validated curriculum, integrating four domains: radiation protection competence, emergency response readiness, psychological resilience and willingness to respond, and organizational well-being. It is advanced as a normative, testable proposition to guide educational reform, local protocol development, and future empirical evaluation.

## Editorial

European nuclear power plants operate under a mature regulatory framework that tightly structures occupational radiation protection for all categories of exposed workers. The International Atomic Energy Agency (IAEA) Safety Standards on occupational radiation protection emphasize dose optimization, monitoring, and risk-based work organization as cornerstones of safe plant operation [1]. Directive 2013/59/Euratom further requires Member States to ensure that the operational protection of exposed workers is based on the optimization of radiation protection and that training appropriate to specific duties is systematically provided [2].

In practice, however, these frameworks speak the language of physics, dosimetry, and facility engineering primarily, with comparatively little attention to the clinical, psychosocial, and educational dimensions of nursing work in nuclear installations. Occupational health nurses in nuclear power plants operate at the interface where regulatory compliance, routine health surveillance, on-site first aid, and emergency response converge, yet their role is rarely described in normative documents beyond generic references to medical services. This conceptual under-specification risks rendering nursing contributions invisible in policy while leaving key competency expectations implicit and therefore unevenly addressed in professional education.

Nuclear utilities typically maintain an internal occupational health and first-aid function staffed by nurses and occupational physicians, often organized on a shift basis to cover 24/7 operations. These nurses manage routine health surveillance of radiation workers, fitness-for-duty assessments, follow-up of minor injuries, and first-response care for acute events pending transfer to external emergency services. During outage periods, when large numbers of contractors are present and collective doses may increase, nurses also contribute to health promotion, occupational risk communication, and coordination with radiation protection teams.

Despite this centrality, the educational profile of occupational health nurses assigned to nuclear plants is usually defined by generic occupational or public health curricula rather than radiation-specific competencies. Available training offerings suggest that, while some programs incorporate modules on ionizing radiation and occupational risk, these remain largely theoretical and disconnected from the operational realities of nuclear power plant work organization, radiological zoning, or emergency procedures for abnormal events. Where this gap has been measured, it is stark: in a multicentre study of 594 emergency nurses, the overwhelming majority demonstrated poor knowledge of radiation protection, radiation health effects, and decontamination, and most reported no prior training in medical response to radiation emergencies [3]. The result is a professional group whose responsibilities are critical for compliance and worker safety, but whose preparation for high-radiation-risk environments often depends on ad hoc, employer-based training rather than structured, nurse-centered educational pathways.

Research on medical personnel’s willingness to engage in nuclear or radiological emergency response highlights a complex interplay between technical knowledge, perceived efficacy, anxiety, and social norms. A recent cross-sectional survey of 378 staff across four university hospitals in Kyushu, Japan, found that willingness to participate in nuclear disaster response was shaped more by psychological and occupational perceptions than by demographic factors or prior disaster experience: interest in radiological and nuclear emergencies, incentives, family understanding, and occupational norms were positively associated with willingness, whereas anxiety was negatively associated [4]. This pattern suggests that preparedness interventions cannot rely on knowledge transfer alone but must also engage motivation, institutional support, and the family and social context in which nurses weigh their readiness to respond.

For nurses working inside, or in close partnership with, nuclear power plants, fear of radiation exposure, the stigma associated with “nuclear work,” and concerns about family reactions may further complicate willingness to participate in on-site emergency response or off-site triage operations. Evidence from occupational radiation exposure studies reinforces this concern: in a large cross-sectional study of medical radiation staff, long-term, low-dose exposure was associated with high levels of job stress and burnout-affecting roughly half and nearly two-thirds of those surveyed, respectively-with job stress itself emerging as the principal risk factor for burnout [5]. These findings underline that preparedness in nuclear settings cannot be reduced to technical drills or knowledge tests: it must also address the psychological, social, and ethical dimensions of nurses’ engagement with radiological risk.

The organizational environments of nuclear power plants and of neighboring referral hospitals tasked with off-site emergency response impose distinctive demands on nurses’ well-being. Working in controlled or supervised areas, complying with strict access and decontamination procedures, and integrating clinical judgment with radiation protection constraints may contribute to cognitive load and moral tension, particularly during abnormal events. More broadly, experience from other high-technology, high-risk clinical settings suggests that cumulative stressors can amplify concerns about personal safety, family exposure, and professional responsibility.

In nuclear power plants, routine operations may foster a sense of controlled, low-risk stability, but rare, high-impact scenarios-such as loss-of-coolant accidents, external hazards, or malicious acts-remain cognitively and emotionally salient, shaping perceptions of the job even when never experienced directly. Without explicit organizational strategies that integrate radiation protection with psychological support, peer debriefing, and recognition of the emotional labor associated with nuclear risk, nurses may internalize anxiety and stigma in ways that undermine retention, performance, and readiness to respond when needed. An occupational health strategy that treats well-being-not only dose metrics-as a core safety parameter is therefore warranted.

Countries that currently operate nuclear power plants or are managing long-term decommissioning, such as France, Sweden, Finland, Belgium, the United Kingdom, and Germany, share a common regulatory substrate in the Euratom Basic Safety Standards, yet differ markedly in how nursing and occupational health services are integrated into nuclear safety frameworks [2]. National guides on medical response to nuclear or radiological emergencies typically delineate roles for emergency physicians, radiological protection teams, and hospital coordination, but provide limited detail on occupational health nurses within the plant perimeter. Where utilities have invested in digital occupational health solutions for nuclear workers-such as remote monitoring and on-site eHealth services-these innovations again tend to foreground physicians and corporate health providers more than nursing.

At the European and international level, hospital preparedness tools have increasingly been adapted to chemical, biological, radiological, and nuclear (CBRN) contexts-including radiological and nuclear scenarios-through simulation-based workshops and table-top exercises. However, participation is often multidisciplinary and non-specific to occupational health nursing, with limited translation into structured competencies or credentialed training pathways for nurses embedded in nuclear installations. The picture that emerges is one of robust technical and regulatory guidance, but fragmented and locally variable approaches to preparing and supporting the occupational health nurses who must operationalize that guidance at the worker level.

In light of these gaps, we propose a heuristic training framework for occupational health nurses working in or with European nuclear power plants-to be read as a practice-oriented, testable model grounded in the foregoing analysis rather than as an empirically validated curriculum. The framework assumes that radiation protection requirements, CBRN preparedness initiatives, and occupational health nursing standards must be integrated into a coherent educational trajectory spanning initial specialization and continuing professional development (CPD). It distinguishes four domains: (1) radiation protection competence; (2) emergency response readiness; (3) psychological resilience and willingness to respond; and (4) organizational well-being and safety culture.

Table 1 outlines how these domains could translate into expected competencies and educational strategies. The model is intentionally generic with respect to national regulations, so that it can be adapted to different legal transpositions of Directive 2013/59/Euratom and related national laws governing occupational exposure and nuclear safety [2]. It is also deliberately framed in action-oriented terms to promote alignment between educational content, plant-level procedures, and broader CBRN preparedness initiatives led by health authorities and international organizations.

**Table 1:** Heuristic training framework (proposed model) for occupational health nurses in European nuclear power plants Authors’ own elaboration. The model is heuristic; its elements require local adaptation, piloting, and rigorous evaluation before any incorporation into formal curricula or accreditation frameworks. CBRN: chemical, biological, radiological, and nuclear.

Domain	Expected competencies	Suggested educational strategies
Radiation protection competence	Understand the principles of ionizing radiation, dose limits, and ALARA; interpret individual and collective dose reports; collaborate effectively with radiation protection officers.	Modular training in radiation physics and protection tailored to nursing practice; supervised rotations with radiation protection teams; periodic refresher courses linked to plant outage cycles.
Emergency response readiness	Perform on-site triage for irradiated and potentially contaminated workers; initiate decontamination pathways and life-saving interventions within radiological constraints; coordinate with external emergency services and off-site medical facilities.	Simulation-based exercises integrating plant emergency plans and hospital CBRN protocols; joint drills with fire, security, and radiation protection units; use of standardized algorithms for radiological triage and decontamination.
Psychological resilience and willingness to respond	Recognize and manage fear of radiation, moral distress, and stigma; maintain willingness to participate in on-site and off-site response; support colleagues’ emotional reactions during and after incidents.	Facilitated debriefings following exercises and incidents; reflective practice groups; targeted workshops on risk communication with families and communities; inclusion of willingness-to-respond scenarios in training evaluations.
Organizational well-being and safety culture	Contribute to a just culture around incident reporting; identify and escalate patterns of fatigue, burnout, or stress related to radiation work; participate in multidisciplinary safety committees.	Training in safety culture and human factors; involvement in occupational health risk assessments; co-design of well-being initiatives (e.g., peer support, rota adjustments) attuned to nuclear plant operational cycles.

This table is intended as a heuristic tool to stimulate dialogue between universities, nuclear operators, occupational health services, and regulatory bodies-not as a prescriptive standard. Its elements require local adaptation, piloting, and rigorous evaluation before any incorporation into formal curricula or accreditation frameworks. For practice, integrating the heuristic framework into plant-level policies would mean explicitly defining the role of occupational health nurses in nuclear emergency plans, clarifying their responsibilities in triage, decontamination, and psychological support, and ensuring they have protected time to participate in simulation exercises and safety committees. Such integration could reduce ambiguity during abnormal events, enhance collaboration with radiation protection and security teams, and improve the continuity of care for affected workers across on-site and off-site settings.

For education, the model suggests that specialist occupational health nursing programs and nuclear-related CPD pathways should move beyond generic references to “ionizing radiation” to include structured modules aligned with plant procedures, national CBRN plans, and international guidance. Accreditation bodies could recognize radiation-focused competencies as a distinct element within occupational health nursing, thereby incentivizing universities and professional associations to develop and evaluate tailored curricula.

For research, the framework points to several testable hypotheses: that higher levels of radiation-specific competence and participation in simulation exercises are associated with increased willingness to respond, lower perceived stress, and better safety-culture indicators among occupational health nurses in nuclear plants. Mixed-methods studies combining surveys, qualitative interviews, and observational data from drills could assess the feasibility, acceptability, and impact of implementing the proposed domains and strategies across different national contexts.

This editorial therefore advances a normative proposal rather than a definitive solution: a heuristic training framework that foregrounds occupational health nurses as critical actors in the safety, readiness, and well-being of the European nuclear workforce. Its value will depend not on its elegance on paper, but on the willingness of institutions to translate it into practice and to subject it to rigorous empirical scrutiny.
